# Traumatic Brain Injury Upregulates Phosphodiesterase Expression in the Hippocampus

**DOI:** 10.3389/fnsys.2016.00005

**Published:** 2016-02-05

**Authors:** Nicole M. Wilson, David J. Titus, Anthony A. Oliva, Concepcion Furones, Coleen M. Atkins

**Affiliations:** The Miami Project to Cure Paralysis, Department of Neurological Surgery, University of Miami Miller School of MedicineMiami, FL, USA

**Keywords:** cAMP, CREB, fluid-percussion, hippocampus, inflammation, phosphodiesterase, traumatic brain injury

## Abstract

Traumatic brain injury (TBI) results in significant impairments in hippocampal synaptic plasticity. A molecule critically involved in hippocampal synaptic plasticity, 3′,5′-cyclic adenosine monophosphate, is downregulated in the hippocampus after TBI, but the mechanism that underlies this decrease is unknown. To address this question, we determined whether phosphodiesterase (PDE) expression in the hippocampus is altered by TBI. Young adult male Sprague Dawley rats received sham surgery or moderate parasagittal fluid-percussion brain injury. Animals were analyzed by western blotting for changes in PDE expression levels in the hippocampus. We found that PDE1A levels were significantly increased at 30 min, 1 h and 6 h after TBI. PDE4B2 and 4D2 were also significantly increased at 1, 6, and 24 h after TBI. Additionally, phosphorylation of PDE4A was significantly increased at 6 and 24 h after TBI. No significant changes were observed in levels of PDE1B, 1C, 3A, 8A, or 8B between 30 min to 7 days after TBI. To determine the spatial profile of these increases, we used immunohistochemistry and flow cytometry at 24 h after TBI. PDE1A and phospho-PDE4A localized to neuronal cell bodies. PDE4B2 was expressed in neuronal dendrites, microglia and infiltrating CD11b^+^ immune cells. PDE4D was predominantly found in microglia and infiltrating CD11b^+^ immune cells. To determine if inhibition of PDE4 would improve hippocampal synaptic plasticity deficits after TBI, we treated hippocampal slices with rolipram, a pan-PDE4 inhibitor. Rolipram partially rescued the depression in basal synaptic transmission and converted a decaying form of long-term potentiation (LTP) into long-lasting LTP. Overall, these results identify several possible PDE targets for reducing hippocampal synaptic plasticity deficits and improving cognitive function acutely after TBI.

## Introduction

Every year in the United States an estimated 1.7 million people sustain a traumatic brain injury (TBI) and nearly 70–80% of those who survive report chronic learning and memory deficits (Lew et al., 2006; Faul et al., 2010). The hippocampus is critically involved in learning and memory and is highly susceptible to damage after TBI. Even when not directly damaged, hippocampal atrophy and neuronal loss are often observed in TBI patients (Bigler et al., 1997; Tomaiuolo et al., 2004). This progressive hippocampal atrophy is also observed in experimental models of TBI, and is accompanied by a loss of dentate hilar cells and neurons in the CA3 region as well as synaptic loss (Maxwell et al., 2003; Hall et al., 2005; Scheff et al., 2005; Witgen et al., 2005). In addition to these gross morphological changes, in experimental models of TBI there are deficits in hippocampal basal synaptic transmission and long-term potentiation (LTP; Miyazaki et al., 1992; Titus et al., 2013b). Finding molecular targets to attenuate the damage caused by TBI and improve hippocampal synaptic plasticity is of critical importance to assist the estimated 3–5 million people currently living with cognitive disabilities from TBI in the United States (Langlois et al., 2006; Zaloshnja et al., 2008).

Targeting TBI-induced LTP deficits is a promising strategy for improving learning and memory (Atkins, 2011). During hippocampal LTP and long-term memory formation, 3′,5′-cyclic adenosine monophosphate (cAMP) signaling is critical to activate the transcription factor cAMP-response element binding protein (CREB) and mediate gene transcription required for long-term memory formation (Frey et al., 1993; Bourtchuladze et al., 1994). We have found that in an experimental model of TBI, cAMP levels are depressed and CREB activation is impaired during a learning task (Titus et al., 2013a,b). The exact molecular mechanisms leading to the decrease in cAMP levels and impairments in CREB activation after TBI are unknown. cAMP is synthesized from ATP by adenylyl cyclases (ACs) and cAMP signaling is tightly regulated in discrete spatial-temporal microdomains through hydrolysis by phosphodiesterases (PDEs; Houslay, 2010). Whether cAMP signaling in the hippocampus is decreased after TBI by either changes in ACs or PDEs is unknown and this knowledge could guide the development of therapeutic strategies for treating the cognitive consequences of TBI.

One strategy for improving cAMP signaling is to inhibit PDEs. However, an important consideration is that the PDE superfamily is a large family of enzymes with 11 different PDE families (Bender and Beavo, 2006; Omori and Kotera, 2007). Within each of the PDE families there are multiple subfamilies encoded by individual genes, with several splice variants within each subfamily. Eight of the PDE families hydrolyze cAMP: PDEs 1, 2, 3, 10, and 11 hydrolyze both cAMP and cGMP, and PDEs 4, 7, and 8 hydrolyze only cAMP (Maurice et al., 2014). Of these PDEs, PDE1, 3, 4, 8, and 10 have been found in the hippocampus (Johansson et al., 2012). The PDE1 family consists of three subfamilies, PDE1A, 1B, and 1C, which are encoded by separate genes. PDE1 is commonly referred to as a Ca^2+^/calmodulin-stimulated PDE, and the activity of PDE1 can be enhanced through increases in calcium and calmodulin signaling (Omori and Kotera, 2007; Heckman et al., 2015). The PDE3 family consists of two subfamilies, PDE3A and 3B, which are encoded by two separate genes. While PDE3 hydrolyzes cAMP and cGMP, the ability for PDE3 to hydrolyze cAMP is inhibited in the presence of cGMP (Bender and Beavo, 2006). The PDE8 family is a cAMP-specific PDE that consists of two subfamilies, PDE8A and 8B (Martinez and Gil, 2014). The PDE10 family is another dual-specific PDE which is encoded by one gene, PDE10A (Kelly and Brandon, 2009). Given the wide array of inhibitors that target each of these families, it is important to understand how TBI affects the expression and activity of each these enzymes to target the relevant PDE (Menniti et al., 2006; Spina, 2008; Titus et al., 2015b).

Of the cAMP-degrading PDEs, the most notable one in the context of learning and memory is the PDE4 family (Li et al., 2011; Heckman et al., 2015). The PDE4 family is encoded by four genes, PDE4A, 4B, 4C, and 4D. With the exception of PDE4C, all of these subfamilies are expressed in the brain, immune system and cardiovascular system (Henkel-Tigges and Davis, 1990; Reneerkens et al., 2009). A unique feature of the PDE4 family is that they are further classified into four groups: long, short, super-short, and dead-short isoforms (Titus et al., 2015a). This classification is based on the presence, or absence, of upstream conserved regions (UCR) near the N-terminus, which allow for differential post-translational regulation of these isoforms (Hansen and Zhang, 2015). The PDE4A family has six isoforms: PDE4A1, 4A4/5, 4A7, 4A8, 4A10, and 4A11. The PDE4B family has five isoforms: PDE4B1-5. The PDE4D family has 10 isoforms: 4D1–7, 4D9, 4D10, and 4D11. Identifying the spatial and temporal expression pattern of these PDEs in the hippocampus after brain injury could facilitate the development of a more targeted approach for reducing TBI-induced cognitive deficits.

Targeting cAMP-degrading PDEs acutely after trauma has yielded promising results in experimental models of spinal cord injury and cerebral ischemia (Kato et al., 1995; Schaal et al., 2012). However, pan-PDE inhibitors have broad effects given the widespread expression of PDE isoforms. In the context of experimental TBI, the anti-inflammatory and neuroprotective effects of pan-PDE4 inhibitors are overshadowed by the vasodilation and hemorrhagic effects when administered acutely after trauma (Atkins et al., 2012, 2013). This emphasizes the need for more specific targets when treating the acute phase of TBI. In a previous study, we reported that TBI alters PDE expression in the ipsilateral parietal cortex acutely after trauma, but whether similar changes occur in the hippocampus is unknown (Oliva et al., 2012). Determining which PDEs are upregulated after trauma could provide more specific drug targets for reducing neuronal death, inflammation and atrophy in the hippocampus after TBI. In this study we investigated changes in PDE1, 3, 4, 8, and 10 after TBI using western blot analysis, immunohistochemistry and flow cytometry analysis of the hippocampus.

## Materials and Methods

### Fluid-Percussion Injury Surgery

One hundred adult male Sprague Dawley rats were used in this study (2–3 mos, 300–350 gm, Charles River Laboratories). All experimental procedures were performed in accordance with the NIH *Guide for the Care and Use of Laboratory Animals* and with approval from the University of Miami Animal Care and Use Committee. Animals were anesthetized (3% isoflurane, 70% N_2_O, 30% O_2_, 5 min) and received a 4.8 mm diameter craniotomy at –3.8 mm posterior to bregma and 2.5 mm lateral to the midline over the right parietal cortex. A plastic female Luer Lock adapter (18 gage) was affixed at the craniotomy site with cyanoacrylate and dental cement. Animals were allowed to recover for 12–16 h while fasting with water ad libitum. Animals were re-anesthetized (induction for 5 min with 3% isoflurane, 70% N_2_O, 30% O_2_, maintenance during surgery with 1% isoflurane, 70% N_2_O, 30% O_2_), then intubated, mechanically ventilated (Stoelting) and given rocuronium (10 mg/kg, intra-arterial) and penicillin/benzathine (20,000 IU/kg, intramuscular). Head and body temperature were maintained between 36.6 and 37.2°C using rectal and temporalis muscle thermistors connected to feedback-regulated heating lamps. Physiological parameters (blood *p*O_2_ and *p*CO_2_, blood pH, and mean arterial blood pressure) were monitored via a tail artery catheter and maintained at normal levels throughout the surgery. Animals were prospectively randomized into sham or TBI surgery groups. Brain trauma was produced with a fluid-pulse (16 ms duration, 2.0 ± 0.2 atmospheric pressure) at the craniotomy site. Sham-operated animals received all surgical procedures identical to the TBI animals with the exception of the fluid-pulse. At the end of the surgery, animals received buprenorphine (0.01 mg/kg, subcutaneously). Criteria for exclusion from the study were: mortality, >15% loss of body weight, non-resolving infection at the surgical site, inability to feed or drink, motor paralysis, listlessness, self-mutilation, excessive grooming leading to loss of dermal layers, spontaneous vocalization when touched or poor grooming habits. No animals were removed from the study. To determine the number of animals needed for the study, a power analysis was prospectively performed to detect a 50% difference in PDE protein expression between groups with western blot analysis at 80% power and significance level of 0.05 (Oliva et al., 2012). An *n* value of 6 animals/group was obtained. Investigators were blind to the animal surgery treatment for the electrophysiology analyses.

### Western Blot Analysis

At 30 min, 1 h, 3 h, 6 h, 24 h, or 7 days after TBI or sham surgery, animals were deeply anesthetized (3% isoflurane, 70% N_2_O, 30% O_2_, 5 min) and decapitated (*n* = 6/time point for TBI animals, *n* = 3/time point for sham animals). The ipsilateral hippocampus was rapidly dissected on ice, snap frozen with liquid nitrogen and stored at –80°C. Tissue was homogenized with a Dounce homogenizer (15 s, 4°C) in: 15 mM Tris pH 7.6, 250 mM sucrose, 1 mM MgCl_2_, 1 mM EGTA, 1 mM DTT, 0.5 mM PMSF, 0.1 mM Na_3_VO_4_, 50 mM NaF, 2 mM Na_4_P_2_O_7_, 1.25 μg/ml pepstatin A, 10 μg/ml leupeptin, 25 μg/ml aprotinin, and 1x phosphatase inhibitor cocktail set II (EMD Millipore). Each hippocampus was homogenized in 750 μl of buffer. Total protein was determined using Coomassie Plus assay (Bio-Rad Laboratories). Homogenates were boiled with sample buffer (9 min, 95°C). Equal amounts of protein per lane (60 μg/sample) were electrophoresed on 12.5% SDS-PAGE gels. Proteins were transferred to Immobilon-P membranes (EMD Millipore) and membranes were incubated with the following primary antibodies: β-actin (AC-15, 1:10,000, Sigma–Aldrich), PDE1A (sc-50480, 1:4,000, Santa Cruz Biotechnology), PDE1B (ab14600, 1:500, Abcam; Giachini et al., 2011), PDE1C (sc-67323, 1:500, Santa Cruz Biotechnology; Haering et al., 2015), PDE3A (sc-20792, 1:250, Santa Cruz Biotechnology; Soler et al., 2015), phospho-PDE4A (GTX14610, 1:2,000, GeneTex), PDE4A5 (ab42094, 1:2,000, Abcam; Carito et al., 2012), PDE4A8 (GTX14606, 1:1,000, GeneTex), PDE4B (sc-25812, 1:500, Santa Cruz Biotechnology; Suhasini et al., 2015), PDE4D (sc-25814, 1:500, Santa Cruz Biotechnology; Kunal et al., 2012), phospho-PDE4D (ab59212, 1:1,000, Abcam), PDE8A (sc-30059, 1:500, Santa Cruz Biotechnology; Dong et al., 2010), PDE8B (sc-17234, 1:500, Santa Cruz Biotechnology; Shimizu-Albergine et al., 2012), and PDE10A (sc-67298, 1:250, Santa Cruz Biotechnology; Giralt et al., 2013). These antibodies were chosen based on previously published studies and resulted in bands that corresponded to the appropriate, apparent molecular weights. Identification of specific PDE isoforms was based on known molecular weights. Secondary antibodies conjugated to horseradish peroxidase were used for detection (1:1,000, Cell Signaling Technology). Epitopes were visualized with enhanced chemiluminescence or enhanced chemiluminescence plus (GE Healthcare) and x-ray film (Phenix Research Products). Quantification of films was performed using ImageJ 1.48v (NIH). Levels of each protein were normalized to β-actin within each sample and then to the average of sham levels. No significant differences in PDE levels were observed in sham animals at the different survival time points (data not shown), so the three sham animals at each recovery time point were pooled into one sample for analysis to reduce animal numbers. Representative western blots show sham animals taken from 3 and 24 h post-recovery.

### Immunohistochemistry

Animals were deeply anesthetized (3% isoflurane, 70% N_2_O, 30% O_2_, 5 min) at 24 h after sham surgery (*n* = 3) or TBI (*n* = 3) and perfused with saline (75 mL) and then with 4% paraformaldehyde in PBS (350 mL, 4°C). Brains were sectioned with a vibratome (50 μm thick) and free floating sections were blocked with PBS containing 5% normal goat serum, 0.2% fish skin gelatin and 0.3% TX-100. Sections were incubated with the following primary antibodies: PDE1A (sc-50480, 1 μg/ml, Santa Cruz Biotechnology), phospho-PDE4A (GTX14610, 1 μg/ml, GeneTex), PDE4B2 (ABS181, 2 μg/ml, Millipore; Ghosh et al., 2012), PDE4D (ABS22, 2 μg/ml, Millipore; Kuroiwa et al., 2012), MAP2 (M9942, 2 μg/ml, Sigma–Aldrich) and NeuN (MAB377, 4 μg/ml, Millipore). Primary PDE antibodies were selected based on our western blot data demonstrating that these antibodies recognized proteins of the appropriate known molecular weights. Secondary antibodies used were conjugated to Alexa 488, Alexa 546 or Alexa 647 (Invitrogen). Cell nuclei were visualized using Hoechst 33342 (Invitrogen).

Images were obtained with a FluoView FV1000 laser scanning confocal microscope (Olympus America) equipped with a 10X 0.4 NA air objective, 20X 0.85 NA oil-immersion objective and 60X 1.42 NA oil-immersion objective, and an LD laser (405 nm), multi-line argon laser and HeNe(G) laser. Sections from different animals were processed in parallel and at least three sections from each animal were imaged.

### Flow Cytometry

At 24 h after sham surgery (*n* = 6) or TBI (*n* = 6), animals were deeply anesthetized (3% isoflurane, 70% N_2_O, 30% O_2_, 5 min) and transcardially perfused with PBS (120 mL, 4°C). The ipsilateral hippocampus was dissected at 4°C. Tissue was mechanically dissociated into single cell suspension and cells were labeled with CD45 Alexa 647 (202212, 1.25 μg/ml, BioLegend) and CD11b v450 (53-4321-80, 1 μg/ml, eBioscience). Dead cells were excluded using LIVE/DEAD Fixable Near-IR dead cell stain (L10119, 1 μl/ml, Life Technologies). Cells were fixed and permeabilized with BD Cytofix/Cytoperm Fixation/Permeabilization kit (554714, BD Biosciences). Cells were intracellularly labeled with phospho-PDE4A (GTX14610, 2 μg/ml, GeneTex), PDE4B2 (ABS181, 2 μg/ml, EMD Millipore) or PDE4D (sc-25814, 2 μg/ml, Santa Cruz Biotechnology). PDE staining was detected with PE-conjugated secondary antibodies (12-4739-81, 10 μg/ml, eBioscience). Flow cytometry data was acquired on a BD LSR II flow cytometer with four emission lasers at 407, 488, 532, and 640 nm. Data collection was performed using BD FACSDiva 8.0.1 (BD Biosciences) and analyzed with Kaluza 1.2 software (Beckman Coulter).

### Electrophysiology

At 24 h after sham surgery (*n* = 13) or TBI (*n* = 15), animals were deeply anesthetized (3% isoflurane, 70% N_2_O, 30% O_2_, 5 min) and then decapitated. The ipsilateral hippocampus was dissected and sliced with a vibratome at 4°C (Leica Microsystems). Slices from the middle third of the hippocampus were collected (400 μm thick) in artificial cerebral spinal fluid (aCSF): 125 mM NaCl, 2.5 mM KCl, 1.25 mM NaH_2_PO_4_, 25 mM NaHCO_3_, 10 mM D-glucose, 2 mM CaCl_2_, 1 mM MgCl_2_ saturated with 95% O_2_/5% CO_2_. Slices recovered at room temperature for at least 60 min. Slices were transferred to a submerged recording chamber and perfused at 2.5–3 ml/min with aCSF at 31°C (Warner Instruments). Field excitatory postsynaptic potentials (fEPSPs) were recorded from the stratum radiatum of area CA1 with glass electrodes filled with 2 M NaCl (1–3 MΩ). The Schaffer collateral pathway was stimulated with a platinum-iridium concentric bipolar electrode (tip diameter 25 μm, FHC). Electrophysiological responses were recorded using a Multiclamp 700B amplifier (Axon Instruments) and pClamp 10.4 software (Axon Instruments). Recordings were low-pass filtered at 2 kHz and digitized at 20 kHz (Digidata 1440A, Molecular Devices). Input–output (I–O) curves were generated with stepwise current increases from 20 to 180 μA. Paired-pulse facilitation (PPF) was measured with 50–250 ms stimulation intervals, delivered at a current intensity of 40–50% of the maximum fEPSP. For baseline responses prior to LTP induction, fEPSPs were recorded at a current intensity of 40–50% of the maximum fEPSP, delivered at 0.033 Hz for at least 20 min. Long-term potentiation (LTP) was induced with high frequency stimulation (HFS) of 100 Hz for 1 s at the current intensity used for baseline stimulation. Rolipram (3 μM) or vehicle (0.3% DMSO) were bath applied in aCSF beginning 10 min prior to LTP induction and for 30 min after tetanization. The amount of depolarization during tetanization was analyzed by integrating the entire HFS response (total) or integrating the last 50 ms of depolarization (steady-state; Klann et al., 1998). Synaptic fatigue was calculated by normalizing each fEPSP during tetanization to the first fEPSP of the HFS (Rutten et al., 2008b).

### Data Analysis

Statistical comparisons were made using GraphPad Prism 6.05 or SigmaPlot 12.0 software. Western blot data and tetanization responses were analyzed using a one-way ANOVA and Tukey’s HSD correction for multiple comparisons. Flow cytometry data was analyzed using an unpaired Student’s *t*-test. I–O responses, PPF, and LTP data were analyzed using a repeated measures two-way ANOVA and Tukey’s HSD correction for multiple comparisons. Significance was designated at *p* < 0.05. Results presented are mean ± SEM.

## Results

**FIGURE 1:**
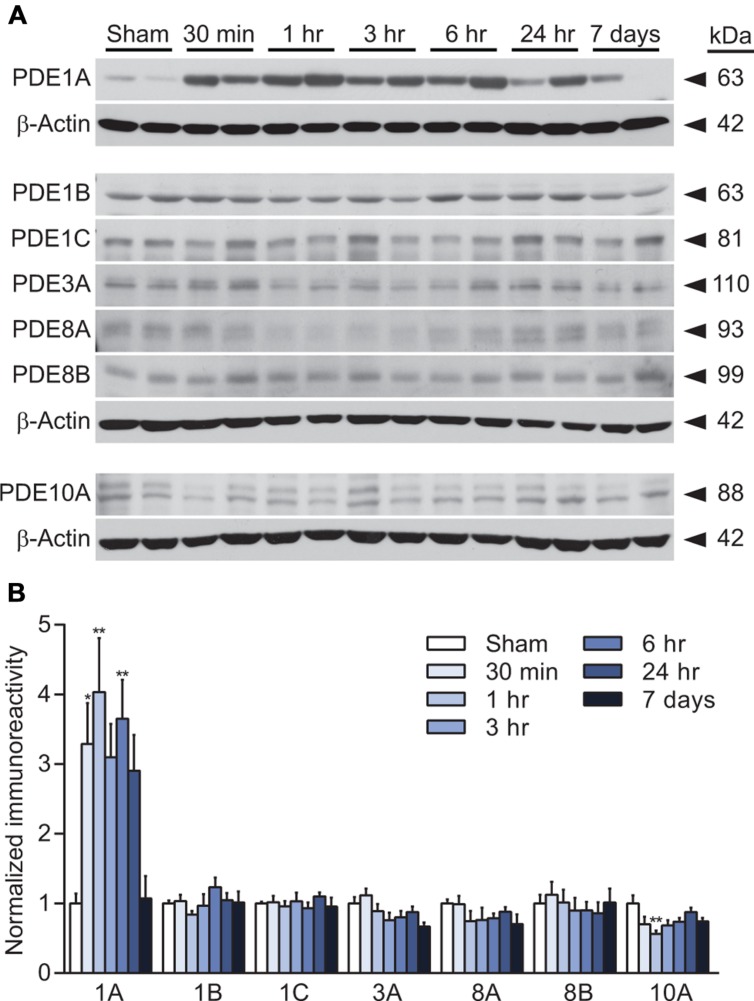
**PDE expression changes after TBI in the hippocampus. (A,C,E)** Representative western blots of the ipsilateral hippocampus analyzed for PDE1A, 1B, 1C, 3A, 8A, 8B, and 10A levels. Each of the corresponding β-actin western blots are shown below the PDE western blots. **(B,D,F)** Densitometry results. Significant increases in PDE1A were observed at 30 min, 1 h and 6 h post-injury. PDE10A decreased at 1 h post-injury. *n* = 6/group, ^∗^*p* < 0.05, ^∗∗^*p* < 0.01 vs. Sham; one-way ANOVA with Tukey’s HSD correction for multiple comparisons.

We have previously reported that cAMP levels are decreased in the hippocampus from 15 min to 4 h after TBI (Atkins et al., 2007). To determine whether this decrease is associated with changes in levels of PDE, the enzyme that degrades cAMP, we evaluated the hippocampus by western blot analysis for PDE expression. We chose to evaluate PDE1, 3, 4, 8, and 10 since these are either cAMP-specific or degrade both cAMP and cGMP and are found in the brain or inflammatory cells that could infiltrate the brain after injury (Boswell-Smith et al., 2006). At 30 min, 1 h, 3 h, 6 h, 24 h, or 7 days after moderate parasagittal fluid-percussion brain injury or sham surgery, animals were analyzed by western blotting for changes in PDE expression in the ipsilateral, injured hippocampus (**Figure 1**). We found that PDE1A was significantly increased as early as 30 min after TBI, and remained elevated at 1 and 6 h post-injury. PDE1B and 1C levels were unchanged after TBI between 30 min to 7 days post-injury. No significant changes in expression were also observed for PDE3A, 8A, or 8B. PDE10A levels were unchanged with the exception of a small decrease at 1 h post-injury.

**FIGURE 2:**
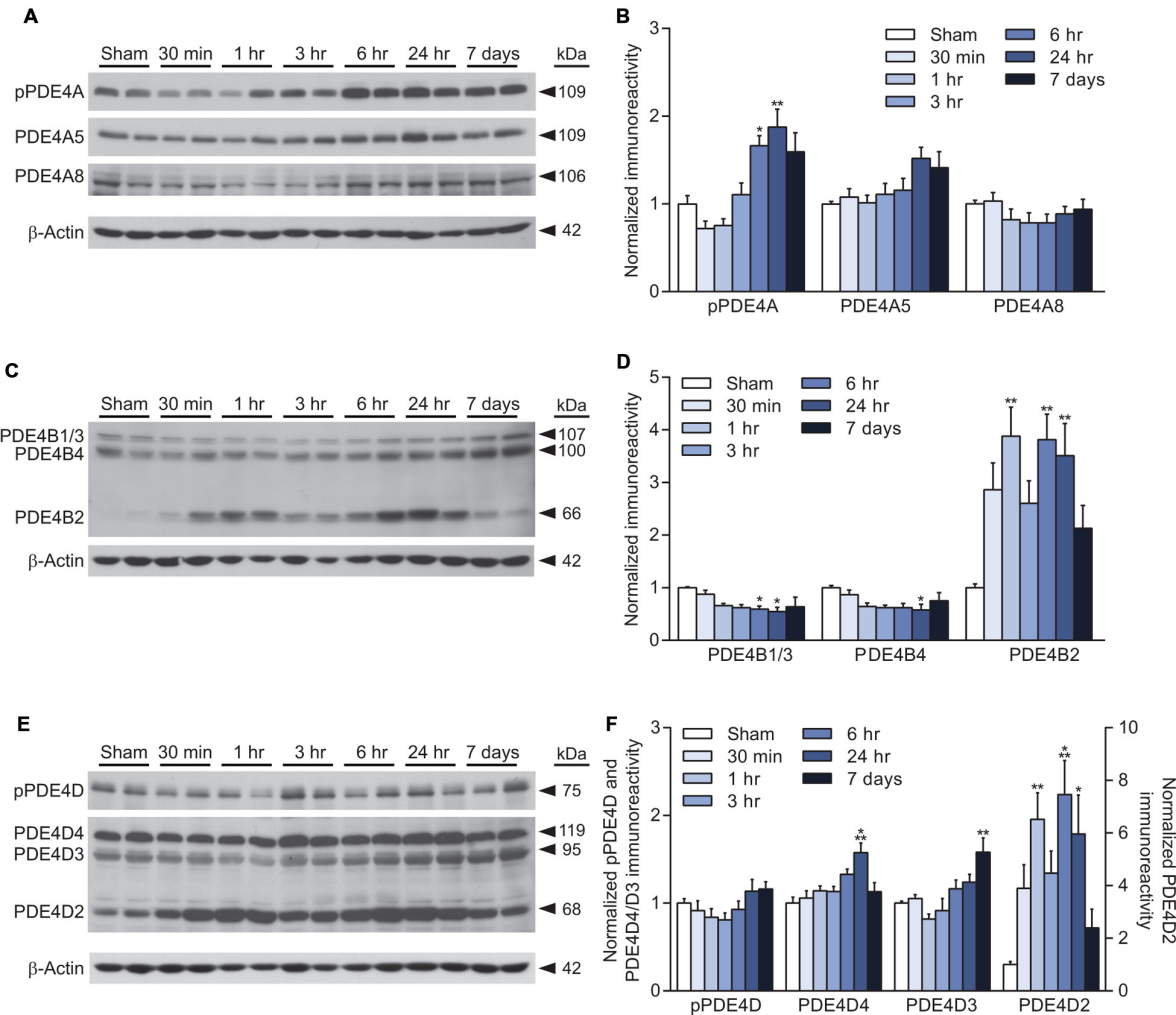
**Increased PDE4B2 and 4D2 expression in the hippocampus after TBI. (A,C,E)** Representative western blots of the ipsilateral hippocampus analyzed for changes in PDE4A, 4B, and 4D. **(B,D,F)** Densitometric analysis. Significant increases in phospho-PDE4A, and total levels of PDE4B2, 4D4, 4D3, and 4D2 were observed. Levels of PDE4B1/3 and 4B4 decreased after TBI. *n* = 6/group, ^∗^*p* < 0.05, ^∗∗^*p* < 0.01, ^∗∗∗^*p* < 0.001 vs. Sham; one-way ANOVA with Tukey’s HSD correction for multiple comparisons.

PDE4 is the major cAMP-degrading enzyme in the brain and the predominant PDE in inflammatory cells (Boswell-Smith et al., 2006). PDE4A, 4B, and 4D are present in the brain, whereas PDE4C is expressed only at very low levels in the brain (Perez-Torres et al., 2000; Lakics et al., 2010; Johansson et al., 2012). PDE4A and 4D are regulated by phosphorylation, and we found that phosphorylation of PDE4A, but not 4D, was significantly increased at 6 and 24 h after TBI (**Figures 2A,E**). Total levels of both PDE4A5 and 4A8 were unaltered after TBI (**Figures 2A,B**). Levels of PDE4B1/3 were modestly, but significantly decreased at 6 and 24 h after TBI (**Figures 2C,D**). Similarly, PDE4B4 was decreased at 24 h after injury. In contrast, both PDE4B2 and 4D2 were significantly increased at 1, 6, and 24 h after TBI (**Figures 2D,F**). PDE4D4 and 4D3 were also elevated at 24 h and 7 days post-injury, respectively (**Figures 2E,F**).

**FIGURE 3:**
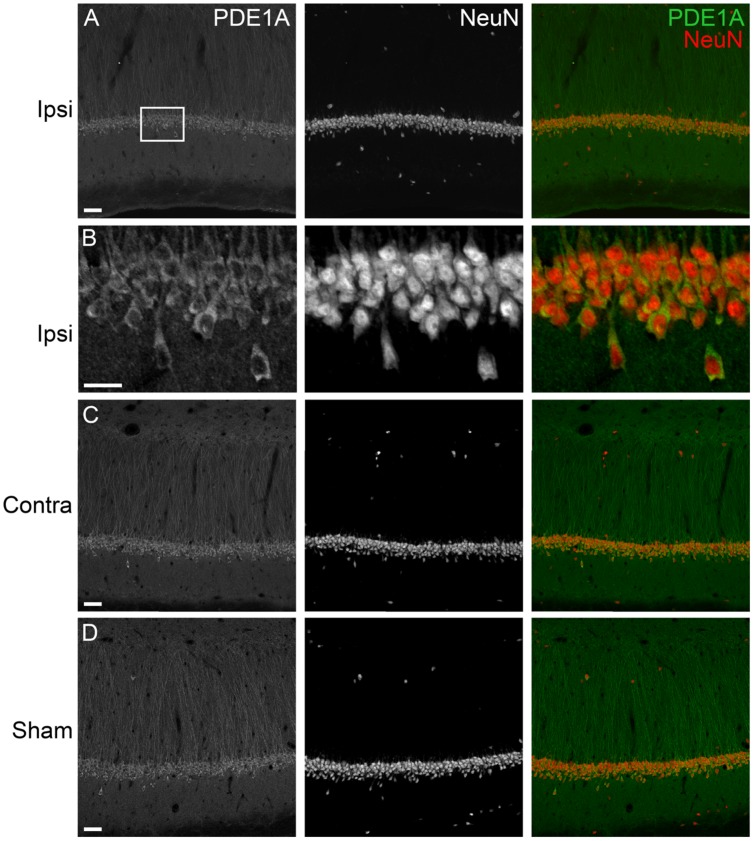
**Immunohistochemistry of PDE1A in the hippocampus. (A)** The ipsilateral hippocampus was immunostained for PDE1A at 24 h after TBI. PDE1A (*green*) colocalized to NeuN-positive cells (*red*) in area CA1. Scale bar 50 μm. **(B)** Higher magnification of the ipsilateral CA1 pyramidal cell layer (*box*). Scale bar 20 μm. **(C)** Expression of PDE1A in the contralateral hippocampus in area CA1. Scale bar 50 μm. **(D)** PDE1A immunohistochemistry in area CA1 from a sham animal. Scale bar 50 μm.

**FIGURE 4:**
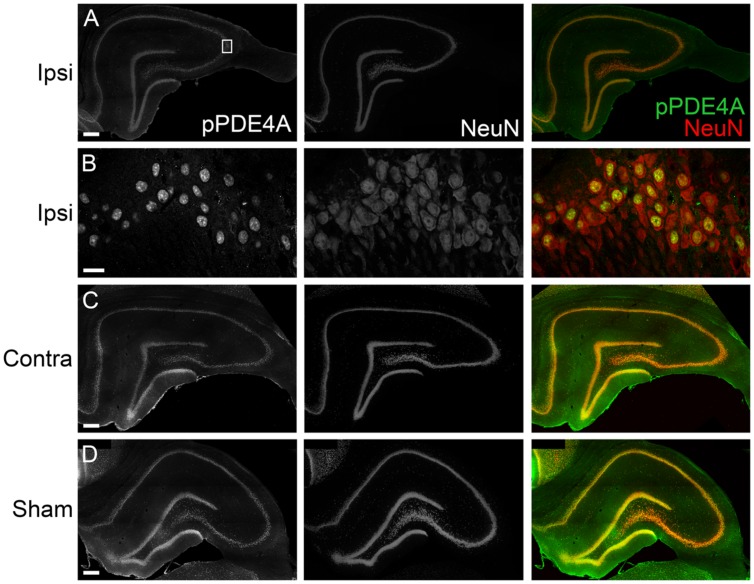
**Immunohistochemistry of phospho-PDE4A in the hippocampus. (A)** At 24 h after TBI, phosphorylated PDE4A (*green*) was found throughout all subregions of the ipsilateral hippocampus and colocalized with NeuN-positive cells (*red*). Scale bar 100 μm. **(B)** Higher magnification of the ipsilateral CA3 pyramidal cell layer (*box*). Scale bar 20 μm. **(C)** Expression of phospho-PDE4A in the contralateral hippocampus. Scale bar 100 μm. **(D)** Phospho-PDE4A immunohistochemistry in the sham, non-injured hippocampus. Scale bar 100 μm.

**FIGURE 5:**
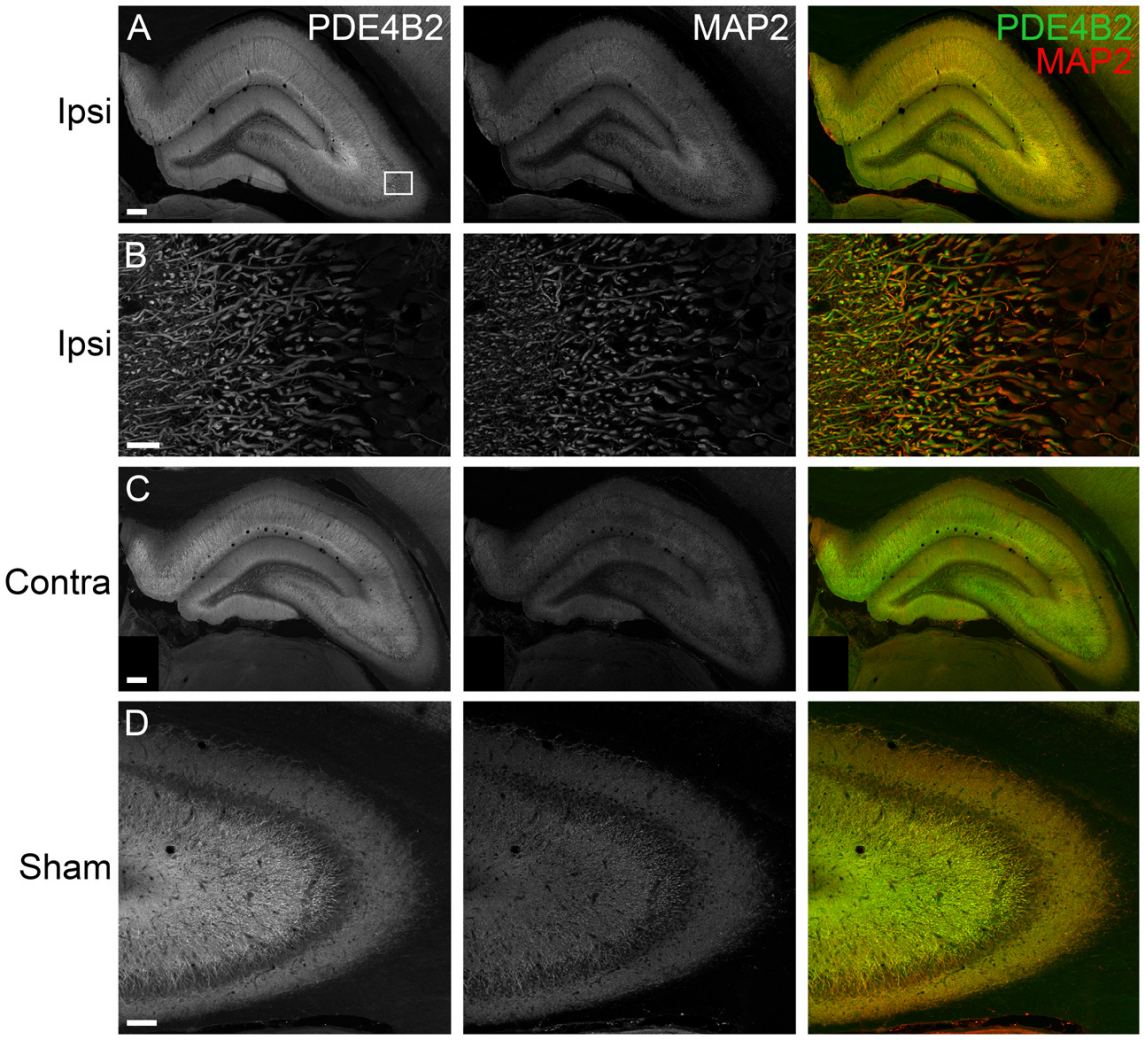
**Immunohistochemistry of PDE4B2 in the hippocampus at 24 h after TBI. (A)** PDE4B2 (*green*) colocalized to MAP2-positive dendrites (*red*) throughout all subregions of the ipsilateral hippocampus. Scale bar 200 μm. **(B)** Higher magnification of the ipsilateral CA3 region (*box*). Scale bar 20 μm. **(C)** Expression of PDE4B2 in dendrites in the contralateral hippocampus. Scale bar 200 μm. **(D)** PDE4B2 immunohistochemistry in area CA3 from a sham animal. Scale bar 100 μm.

**FIGURE 6:**
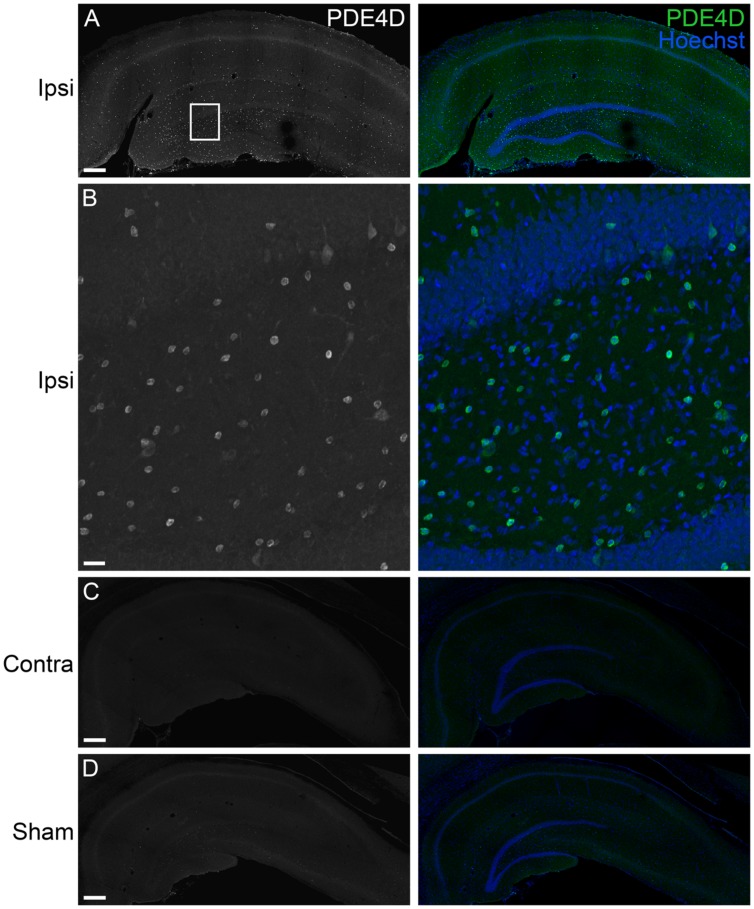
**Immunohistochemistry of PDE4D in the hippocampus at 24 h after TBI. (A)** PDE4D (*green*) was found in scattered cells throughout all regions of the ipsilateral hippocampus. Hoescht counterstaining was used to identify the hippocampal pyramidal cell layers (blue). Scale bar 250 μm. **(B)** Higher magnification of the ipsilateral dentate gyrus (*box*). Scale bar 20 μm. **(C)** Expression of PDE4D was at very low levels in the contralateral hippocampus. Scale bar 250 μm. **(D)** PDE4D was present at very low levels in the sham, non-injured hippocampus. Scale bar 250 μm.

To determine the localization of these changes, we performed immunohistochemistry at 24 h after TBI for PDE1A, phospho-PDE4A, PDE4B2, and PDE4D4, the isoforms that showed the largest changes in expression after TBI. PDE1A was found in nuclei and neurites of neurons (**Figure 3**). Phospho-PDE4A localized predominately to neuronal nuclei (**Figure 4**). In contrast, PDE4B2 was expressed in dendrites as determined by co-localization with MAP2, but not in the cell bodies or nuclei (**Figure 5**). PDE4D immunoreactivity was present in scattered cells throughout the hippocampus, suggestive of immune cell infiltration (**Figure 6**).

**FIGURE 7:**
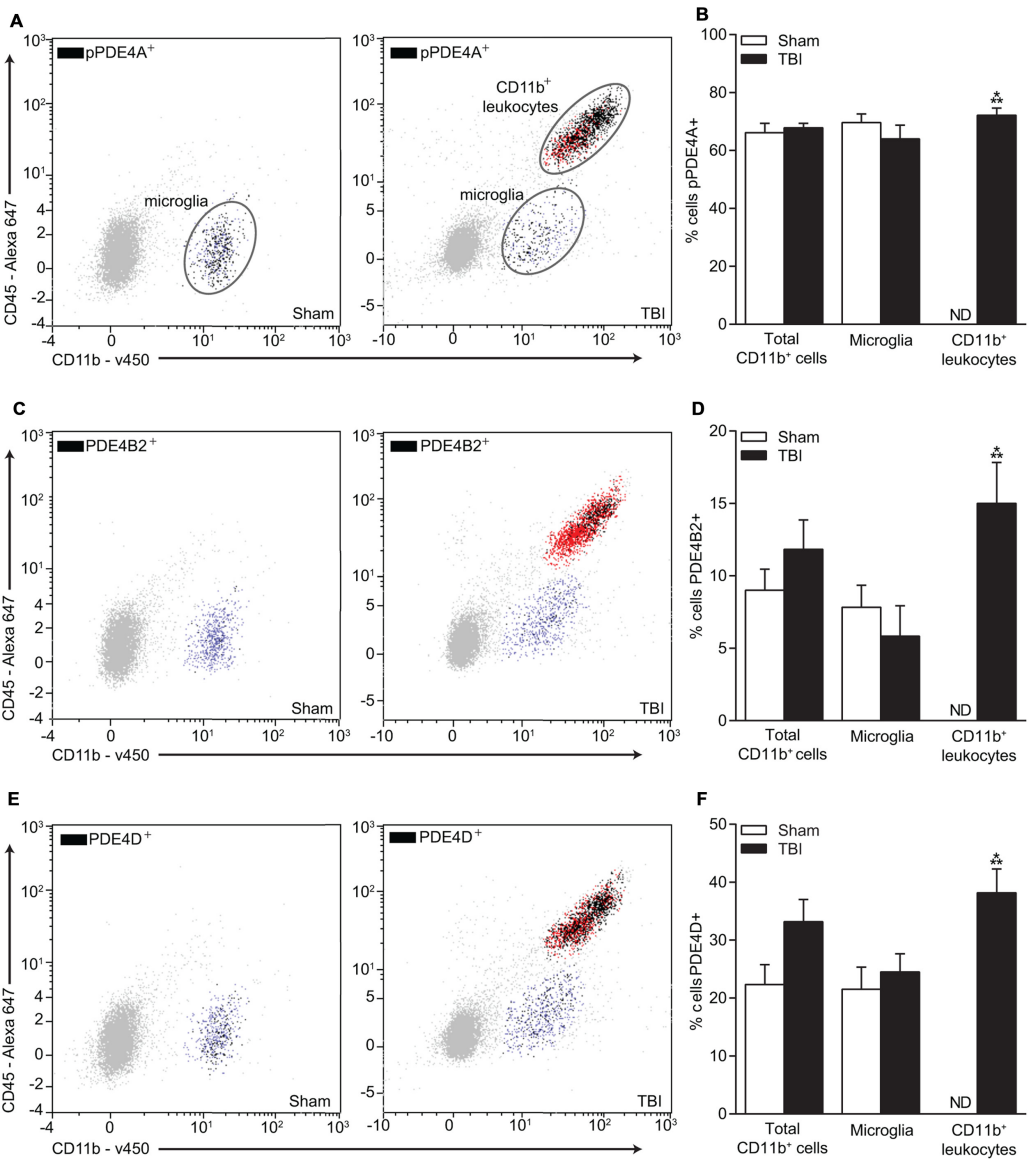
**Flow cytometry of microglia and infiltrating CD11b^+^ immune cells and co-localization with phospho-PDE4A, PDE4B2, and 4D. (A)** Phospho-PDE4A, **(C)** PDE4B2, and **(E)** PDE4D expression were localized to microglia and infiltrating immune cells in the injured hippocampus at 24 h post-injury. There was a significant increase in **(B)** phospho-PDE4A, **(D)** PDE4B2 and **(F)** PDE4D-positive infiltrating CD11b^+^/CD45^+^ immune cells. *n* = 6/group, ^∗∗∗^*p* < 0.001 vs. Sham; unpaired Student’s *t*-test.

To more definitively determine the cellular expression of PDE4D, we performed flow cytometry (**Figure 7**). We also selected PDE4B2 and phospho-PDE4A for analysis since these were also significantly elevated in the injured hippocampus at 24 h post-TBI and have been identified in immune cells (Shepherd et al., 2004; Ghosh et al., 2012). Phospho-PDE4A, PDE4B2, and 4D were localized to microglia and infiltrating CD11b^+^/CD45^+^ immune cells in the hippocampus at 24 h post-injury.

**FIGURE 8:**
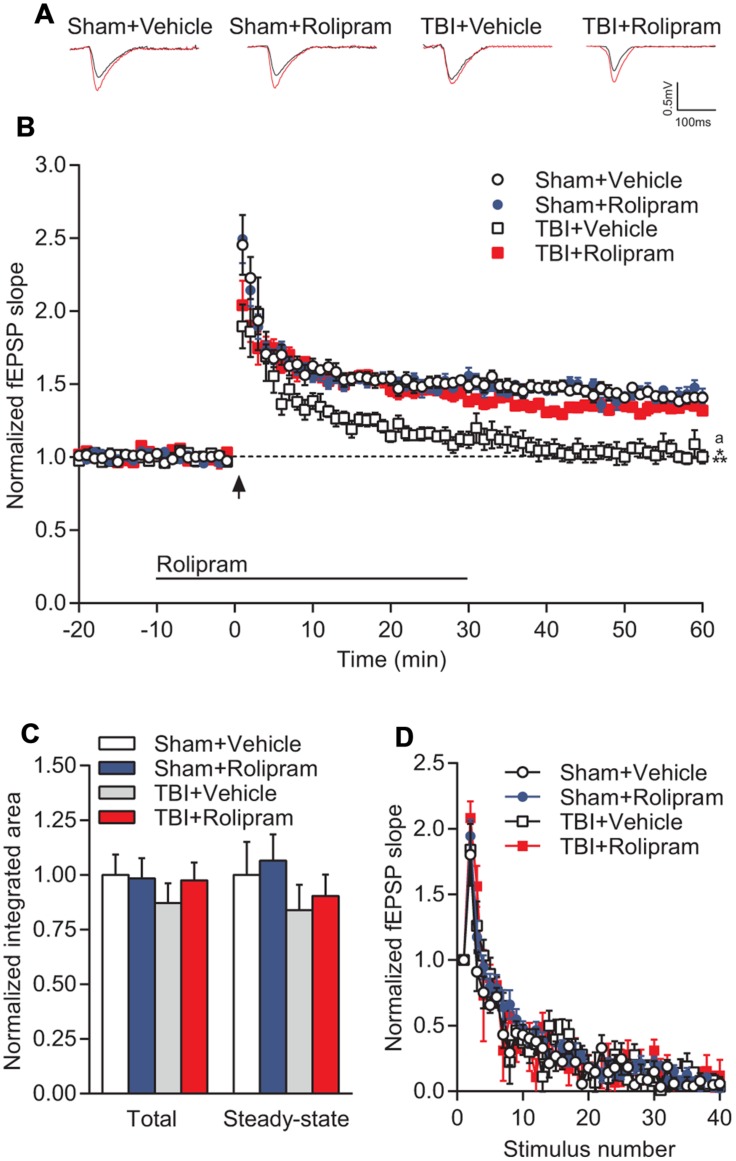
**PDE4 inhibition significantly rescued TBI-induced LTP deficits in area CA1 of the hippocampus. (A)** Representative traces of the fEPSP before (*black*) and 60 min after (*red*) tetanization. **(B)** fEPSP slopes were normalized to baseline prior to LTP induction. Rolipram (3 μM) or vehicle (0.3% DMSO) were bath applied 10 min prior to and for 30 min after tetanization (*bar*). Hippocampal LTP was induced with 1 × 100 Hz tetanization, 1 s long (*arrow*). **(C)** Total and steady-state depolarization levels during tetanization. **(D)** Synaptic fatigue during tetanization. Sham + Vehicle *n* = 10 slices/9 animals, Sham + Rolipram *n* = 14 slices/12 animals, TBI+Vehicle *n* = 8 slices/7 animals, TBI+Rolipram *n* = 8 slices/8 animals; ^∗∗∗^*p* < 0.001 vs. Sham + Vehicle or Sham + Rolipram, ^a^*p* < 0.05 vs. TBI + Rolipram; repeated-measures two-way ANOVA with Tukey’s HSD correction for multiple comparisons.

**FIGURE 9:**
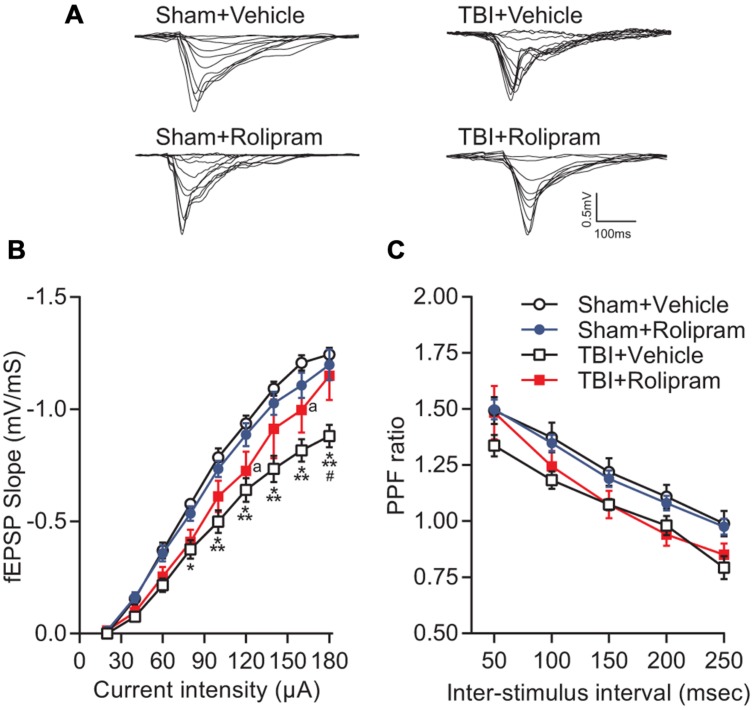
**Effects of rolipram on hippocampal synaptic transmission at 24 h post-TBI. (A)** Representative traces of the fEPSP evoked during input–output (I–O) responses in stratum radiatum of area CA1 with Schaffer collateral stimulation. **(B)** The I–O curve was significantly shifted downward in hippocampal slices from TBI animals as compared to sham animals. Rolipram (3 μM) significantly enhanced the fEPSP slope in slices from TBI animals. ^∗^*p* < 0.05, ^∗∗∗^*p* < 0.001 vs. Sham + Vehicle, ^#^*p* < 0.01 vs. TBI + Rolipram, ^a^*p* < 0.05 vs. Sham + Vehicle. **(C)** A decrease in PPF was observed in TBI + Vehicle-treated slices (*p* < 0.05 TBI + Vehicle vs. Sham + Vehicle) that was not significant in TBI + Rolipram-treated slices. Data represent the ratio of the second fEPSP slope to the first fEPSP slope. Sham + Vehicle *n* = 10 slices/9 animals, Sham + Rolipram *n* = 14 slices/12 animals, TBI+Vehicle *n* = 8 slices/7 animals, TBI + Rolipram *n* = 8 slices/8 animals; repeated-measures two-way ANOVA with Tukey’s HSD correction for multiple comparisons.

Next, to determine if upregulation of PDE4 expression is involved in hippocampal synaptic plasticity deficits after TBI, we assessed whether administration of a pan-PDE4 inhibitor would rescue the deficits in hippocampal LTP induced by TBI. Hippocampal LTP in area CA1 induced with a single tetanus was significantly impaired in slices from TBI animals (**Figures 8A,B**). Rolipram (3 μM) applied to the hippocampal slices rescued the deficits in LTP expression [time × animal treatment interaction *F*_(177,2115)_ = 1.60, *p* < 0.001; **Figures 8A,B**]. Both total and steady-state depolarization levels as well as synaptic fatigue during tetanization were comparable between all groups, suggesting that the impairment in LTP after TBI was not due to differences in depolarization during the tetanus (**Figures 8C,D**). To evaluate whether changes in basal synaptic transmission were involved in the rescue of hippcampal LTP, we analyzed input–output (I–O) responses. I–O curves were significantly depressed in slices from TBI animals as compared to sham animals [stimulation intensity × animal treatment interaction *F*_(24,288)_ = 2.76, *p* < 0.001]. This depression in basal synaptic transmission was partially rescued with rolipram [main effect of animal treatment *F*_(3,288)_ = 8.12, *p* < 0.001; **Figures 9A,B**]. PPF was depressed after TBI and also partially rescued with rolipram treatment [main effect of animal treatment *F*_(3,144)_ = 3.35, *p* < 0.05; **Figure 9C**]. These results indicate that hippocampal basal synaptic transmission and LTP expression in area CA1 were impaired acutely after TBI and rescued with the pan-PDE4 inhibitor, rolipram.

## Discussion

Downregulation of the cAMP signaling pathway following TBI significantly alters hippocampal synaptic plasticity, however, the underlying mechanisms are unknown. The PDE enzymes play a critical role in the regulation of cAMP levels, and alterations in PDE expression and phosphorylation may be involved in the depression of cAMP levels following TBI. Therefore, in the present study we evaluated whether expression of PDEs such as PDE1, 3, 4, 8, and 10, which are present in the central nervous system and hydrolyze cAMP, were altered acutely after TBI (Bender and Beavo, 2006; Omori and Kotera, 2007; Lakics et al., 2010). While not all PDE isoforms showed altered expression levels, several were significantly changed. PDE1A, 4B2, and 4D2, and phosphorylation of PDE4A were significantly upregulated, whereas PDE4B1/3, 4B4, and 10A levels decreased acutely after TBI. The PDEs 1B, 1C, 3A, 4A5, 4A8, 8A, and 8B, and phospho-PDE4D were unchanged acutely after TBI. Although an *a priori* power analysis was conducted to ensure the sample sizes were sufficient to detect significant changes in PDE levels after TBI, for these particular PDEs the negative results should be interpreted cautiously.

Phosphodiesterases are key enzymes involved in regulating cAMP levels in the central nervous system. Understanding the spatio-temporal alterations in PDE isoform expression levels after injury is important for targeting the relevant isoform as well as treating within the appropriate therapeutic time window. In a previous study, we found that there were significant temporal changes in PDE expression within the ipsilateral cortex acutely after TBI (Oliva et al., 2012). Like the present results found in the injured hippocampus, TBI induced upregulation of PDE1A, 4B2, and 4D2 in the ipsilateral cortex. However, not all PDEs were regulated similarly between the cortex and hippocampus. For example, PDE10A was upregulated in the cortex, but downregulated in the hippocampus acutely after TBI. PDE4A5 and 4A8 were downregulated in the cortex, but unaltered in the hippocampus. Furthermore, both PDE4D4 and 4D3 were upregulated at 24 h after TBI in the hippocampus, but unchanged in the cortex. These discrepancies suggest that developing a therapeutic to target a specific PDE and particular functional outcome requires consideration of both the temporal and spatial regulation of that particular PDE isoform.

The upregulation of PDE1A in the hippocampus after TBI suggests that beyond decreasing cAMP levels, cGMP levels may also be affected by TBI. PDE1 is a family of Ca^2+^/calmodulin-dependent PDEs encoded by three genes (A–C) and involved in the regulation of both cGMP and cAMP (Bender and Beavo, 2006). In the human brain, PDE1A is distributed in the parietal cortex and hippocampus, but at lower levels than PDE1B or 1C (Lakics et al., 2010). In the present study, expression of PDE1A was significantly increased, while PDE1B and 1C were unchanged after TBI. Although the PDE1 family uses both cyclic nucleotides as a substrate, PDE1A displays a higher affinity toward cGMP than cAMP (Bender and Beavo, 2006). Conversely, PDE10A which is also a dual-substrate PDE, was downregulated in the hippocampus. These opposing changes may be involved in the lack of changes in basal cGMP levels reported in the hippocampus after TBI (Temple et al., 2001). However, NMDA-stimulated levels of cGMP are actually increased in the ipsilateral hippocampus after TBI and further studies are required to determine if cGMP-selective PDEs expressed in the brain such as PDE9 are altered by TBI (Temple et al., 2001). Together these results support the potential therapeutic use of PDE1 inhibitors such as vinpocetine, amantadine and caffeine, which have shown promise to improve cognitive impairments in preclinical models of TBI and in humans (Dixon et al., 1999; Li et al., 2008; Amen et al., 2011).

In particular, the PDE4 family was highly regulated in the hippocampus after TBI. PDE4B2, 4D2, 4D3, and 4D4 expression levels and phosphorylation of PDE4A were increased following TBI, while PDE4B1/3 and 4B4 levels were decreased. Due to the similar molecular weights of PDEB1 and B3, we were unable to differentiate which particular isoform decreased after TBI. The changes in PDE4 isoforms were transient with most returning to sham, non-injured levels by 7 days post-injury. The upregulation of PDE4 isoforms correlates loosely with the decrease in cAMP levels and PKA activation after TBI (Atkins et al., 2007). However, a causal link between an upregulation of PDE4 expression and decreased cAMP levels has not been established. Alternatively, changes in AC activity or expression could be involved in the decrease in cAMP levels after TBI. Accordingly, reduction in type 1 AC enzyme levels have been reported within the hippocampus of Alzheimer’s disease patients, aged rodents and after ischemia, and are correlated with impairments in cAMP signal transduction (Araki et al., 1995; Yamamoto et al., 2000; Nagakura et al., 2002; Ramos et al., 2003; Mons et al., 2004). Further studies are needed to determine if AC levels are also altered by TBI or whether the increase in PDE expression after TBI also results in increased PDE activity.

The PDE4 subfamily, like other PDEs, is subject to post-translational modification to allow for rapid feedback in controlling the spatial and temporal dynamics of cAMP signaling (Houslay, 2010). Unlike the other PDE4 isoforms, the long isoforms have a UCR1 domain that contains a PKA phosphorylation site that enhances PDE4 activity (Mackenzie et al., 2002). The PKA phosphorylation site allows for cAMP signaling to negatively feedback on itself through enhanced activation of long PDE4A isoforms. PDE4A5 and 4A8 are both long isoforms with similar molecular weights, which precluded definitively identifying which isoform was increased in phosphorylation after TBI. Phospho-PDE4A localized to both hippocampal neurons and infiltrating CD11b^+^ leukocytes. While this increase in phospho-PDE4A is indicative of an increase in PKA-mediated PDE4A activity, the timing of this change does not correlate with changes seen in cAMP and phospho-PKA, which are depressed in the hippocampus during this time frame (Atkins et al., 2007). However, it is possible that there could be changes in cAMP and PKA signaling in microdomains mediating this effect that were missed with the western blot analysis or immunohistochemistry. Subcellular, compartmentalized signaling changes have been reported when assessing differences between PDE4B and 4D signaling (Blackman et al., 2011). An alternative possibility was that another protein kinase mediated this phosphorylation, such as MAPK-activated protein kinase 2, which has been reported to phosphorylate a serine near the PKA phosphorylation site of PDE4A5 (Mackenzie et al., 2011).

In contrast to the results with phospho-PDE4A, the increase in PDE4B2 and 4D2 expression correlated with the decrease in cAMP levels after TBI (Atkins et al., 2007). Within 1 h after trauma, PDE4B2 and 4D2 were significantly elevated in the injured hippocampus and remained elevated for up to 24 h post-injury. Although the immunohistochemistry results indicated that PDE4B2 and phospho-PDE4A localized to neurons, flow cytometry revealed expression of PDE4B2 and phospho-PDE4A in microglia and infiltrating CD11b^+^/CD45^+^ leukocytes as well. The absence of obvious expression in microglia or infiltrating immune cells with immunohistochemistry suggests that phospho-PDE4A- and PDE4B2-positive immune cells may be present near the contusion site between the parietal cortex and hippocampus which was likely sampled in the western blot analysis. Immunohistochemistry of this damaged area can be hindered by autofluorescence of red blood cells present at the contusion site. These findings are supported by other studies which have found that both isoforms are differentially distributed throughout the brain and also found in the immune system. PDE4B and 4D are present in monocytes, macrophages and neutrophils and regulate pro-inflammatory mediators such as tumor necrosis factor and neutrophil infiltration (Jin and Conti, 2002; Ariga et al., 2004; Shepherd et al., 2004). Interestingly, phospho-PDE4A and PDE4D were expressed in a larger proportion of infiltrating CD11b^+^/CD45^+^ cells as compared to PDE4B2. While the increase in phospho-PDE4A, PDE4B2, and PDE4D in immune cells may contribute to inflammatory signaling after TBI, the upregulation of PDE4B2 in hippocampal dendrites suggests that this molecule may have multiple roles in TBI and may also be involved in learning and memory deficits after TBI.

PDE4 is well known to be involved in hippocampal LTP and long-term memory formation (Navakkode et al., 2004; Rutten et al., 2008a,b; Li et al., 2011). General pan-PDE4 inhibitors such as rolipram reverse learning and memory impairments in animal models of Alzheimer’s disease, psychosis, stress, and ischemia (Henkel-Tigges and Davis, 1990; Imanishi et al., 1997; Bach et al., 1999; Gong et al., 2004; Chen et al., 2010; Wiescholleck and Manahan-Vaughan, 2012; Sierksma et al., 2014). In accordance with these previous studies, we found that bath application of rolipram to hippocampal slices from TBI animals reversed the decay of LTP (Navakkode et al., 2004). However, the dose of rolipram required to rescue hippocampal LTP (3 μM) at 24 h post-injury was threefold greater than the dose sufficient to rescue hippocampal LTP deficits at 2 weeks post-injury (1 μM; Titus et al., 2013a,b). Indeed, 1 μM rolipram did not rescue the deficits in hippocampal LTP at 24 h post-injury (data not shown). Given that expression of most PDE4 isoforms returned to sham, non-injured levels by 7 days post-injury, we speculate that the acute, transient upregulation of PDE4 isoforms at 24 h post-injury resulted in the necessity for a higher dose of rolipram to rescue hippocampal LTP deficits at this acute time point.

Interestingly, rolipram also partially improved the depression of basal synaptic transmission in area CA1 of the hippocampus. Basal synaptic transmission in the hippocampus is mediated primarily through AMPA-type glutamate receptors, which are well known to be regulated by PKA (Man et al., 2007). Trafficking of AMPA-type glutamate receptors into the postsynaptic membrane is increased by PKA phosphorylation of serine 845 on the GluA1 subunit (Oh et al., 2006; Middei et al., 2013). GluA1 levels have been found to be altered in some experimental models of TBI (Schumann et al., 2008; Kharlamov et al., 2011). The observed partial rescue in basal synaptic transmission may possibly be through regulation of AMPA receptor trafficking although further studies are needed to evaluate this potential mechanism.

In summary, we found that in an experimental model of TBI there was an acute upregulation of PDE1A, 4B2, 4D4, 4D3, 4D2, and phosphorylated PDE4A in the hippocampus after injury. Furthermore, we found that PDE4B2 and phospho-PDE4A were localized to neurons, microglia and infiltrating CD11b^+^ leukocytes in the injured hippocampus early after trauma. PDE4D was found predominantly in microglia and infiltrating CD11b^+^/CD45^+^ leukocytes. Rolipram, a pan-PDE4 inhibitor, rescued synaptic plasticity deficits in the hippocampus at 24 h after TBI. Given the role of these PDE4 subfamilies in learning, memory and inflammation, this study highlights new potential targets for reducing damage and improving hippocampal synaptic plasticity after TBI.

## Author Contributions

NW contributed in experimental design, data collection and analysis, and writing of the manuscript. DT contributed in experimental design, data collection and analysis, and writing of the manuscript. AO contributed in experimental design, data collection and analysis, and writing of the manuscript. CF contributed in experimental design, data collection, and writing of the manuscript. CA contributed in experimental design, data analysis, and writing of the manuscript.

## Conflict of Interest Statement

The authors declare that the research was conducted in the absence of any commercial or financial relationships that could be construed as a potential conflict of interest.
